# 研究者发起的临床研究用于支持新药研发面临的挑战

**DOI:** 10.3779/j.issn.1009-3419.2022.102.31

**Published:** 2022-07-20

**Authors:** 桦 白, 抒扬 张

**Affiliations:** 1 100730 北京，北京协和医院科研处 Department of Scientific Research, Peking Union Medical College Hospital, Chinese Academy of Medical Sciences & Peking Union Medical College, Beijing 100730, China; 2 100730 北京，北京协和医院心内科 Department of Cardiology, Peking Union Medical College Hospital, Chinese Academy of Medical Sciences & Peking Union Medical College, Beijing 100730, China

**Keywords:** 研究者发起的临床研究, 新药研发, 质量管理, 伦理审查, Investigator-initiated clinical trials, New drug development, Quality management, Ethical review

## Abstract

在我国开展的大量研究者发起的临床研究（Investigator-initiated clinical trials, IIT），其中部分应该可以为新药研发起到重要的支撑作用。但由于我国IIT尚存在数量多、规模小、质量参差不齐等问题，特别是在方案设计、质量管理以及伦理审查能力等方面与制药企业发起的注册临床试验还存在不小的差距，导致很多IIT还不能用于支持新药研发。因此需要监管部门、申办方、研究机构、伦理委员会和研究者共同提高对于IIT用于支持新药研发作用的认识。只有加强监管，建立有效的质量管理体系、强化对研究者的培训并切实提升伦理审查能力，才能用高质量的研究者发起的临床研究支持新药研发。

## Abstract (en)

A large number of investigator-initiated clinical trials (IIT) were conducted in China, some of them should play an important supporting role in new drug development. Due to the large number, small scale and uneven quality of IIT in China, especially a big gap between the IIT and industry-sponsored trials in terms of protocol design, quality management and ethical review, many IIT can't be used to support the new drug development. Therefore, it is necessary for regulatory authorities, sponsors, research institutions, ethics committees and researchers to improve their understanding of the role of IIT. In order to support the new drug development with high-quality IIT, formulating supervising system, establishing an effective quality management system, enhancing the training of researchers and improving the ability of ethical review should be implemented effectively.

为了在生物医药领域大力推进科技创新和产业化应用，实现生物医药技术惠民，国家先后出台了《关于全面推进卫生与健康科技创新的指导意见》、《关于深化审评审批制度改革鼓励药品医疗器械创新的意见》、《“十四五”生物经济发展规划》等一系列政策。2020年我国开展的新药临床试验已经超过仿制药，占比达到57%，说明我国正在实现从仿制药大国向创新药强国的转变。

近年来，制药企业发起的注册临床试验（industry-sponsored trial, IST）是监管部门批准新药上市的主要依据，但精心设计、良好组织实施的研究者发起的临床研究（investigator-initiated clinical trials, IIT），对于支持新药研发的积极推动作用越来越受到重视。由于IIT的设计、实施及质量与IST存在差距，使得监管部门、企业和研究者在利用IIT支持新药研发时还存在顾虑。进一步了解存在的问题、分析面临的挑战，进而提出解决策略，提高IIT的整体水平，将有助于推动IIT支持新药研发。

## 1 IIT

国家卫生健康委2021年9月9日发布《医疗卫生机构开展研究者发起的临床研究管理办法（试行）》中规定，IIT指医疗卫生机构开展的，以个体或群体（包括医疗健康信息）为研究对象，不以药品医疗器械（含体外诊断试剂）等产品注册为目的，研究疾病的诊断、治疗、康复、预后、病因、预防及健康维护等的活动。由于IST是由企业出资发起，由企业主导，可能更倾向于得出有利于其产品的结论^[1]^，而IIT往往来自于医疗实践中产生的临床问题，主要目标是学术和医疗管理，而不是商业目的，因此IIT可能比IST更为可信，其研究结果和发现不仅仅可以产生学术影响，推动制定医疗实践指南，也可以在新药研发中发挥重要作用。

在www.clinicaltrials.gov网站上搜索2012年-2021年在中国开展的研究类型为Interventional Studies（Clinical Trials）的项目，基金来源于企业的作为IST，基金来源于美国卫生研究院、美国政府、个人、大学、组织的作为IIT。结果发现10年间，IIT从520项增加到1, 673项，占比波动于59.1%-75.3%（图 1），一定程度上反映了在我国IIT正在蓬勃开展的态势。与近年来新药研发的热点集中在肿瘤领域一致，IIT研究中，肿瘤相关研究的占比高达36.3%-48.5%（图 2），且开展较多的适应证多为我国的主要瘤种：肺癌、乳腺癌、食管癌、结直肠癌、胃/胰腺/壶腹部癌^[2]^，说明IIT更贴近临床，主要针对的是我国未被满足的临床需求。

**图 1:**
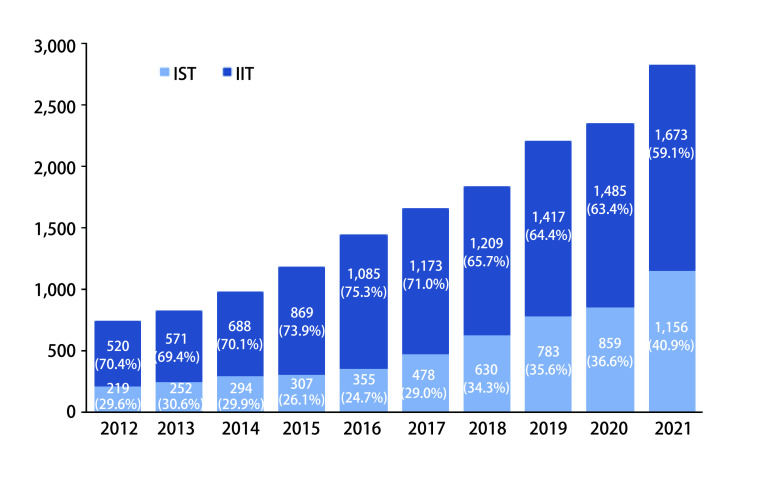
近10年中国在www.clinicaltrials.gov注册的干预性临床研究数量 The amount of interventional studies in China registered at www.clincaltrials.gov in the past 10 years. IST: industry-sponsored trial; IIT: investigator-initiated clinical trials

**图 2:**
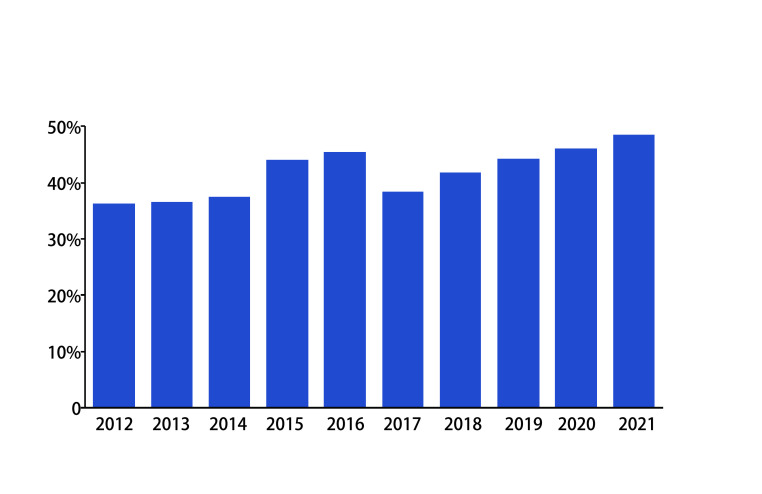
近10年中国在www.clinicaltrials.gov注册的干预性IIT中肿瘤研究占比 Proportion of tumor studies in interventional IIT in China in the past 10 years registered at www.clincaltrials.gov

## 2 IIT用于支持新药研发

虽然在新药研发领域，主要的临床研究类型仍然是IST，但高质量的IIT可以作为直接证据或重要的参考材料用于监管部门新药注册（new drug application, NDA）审评，特别是作为支持批准新增适应证的重要参考^[3]^。例如：欧洲药品质量管理局曾基于一项IIT的结果，批准美罗华扩展适应证用于治疗成人寻常型天疱疮患者^[4]^。在2021年美国食品药品监督管理局（Food and Drug Administration, FDA）基于1项IIT的数据，批准普乐可复与其他免疫抑制性药物联用，预防接受肺移植的成人和儿科患者的器官排斥反应^[5]^。2018年中国国家食品药品监督管理局药品审评中心（Center for Drug Evaluation, CDE）基于3项IIT提供的强有力的证据，批准贝伐珠单抗联合以铂类为基础的化疗方案可用于晚期非鳞状非小细胞肺癌的一线治疗^[6]^。

除了作为NDA的直接证据或参考材料，IIT还能为新药研发提供方向、指导临床试验设计，或者作为基础条件，支持关键注册临床试验的方案设计、受试者人群选择、样本量估算等。在实施双轨制管理的细胞治疗领域，先于IST开展的IIT提供的材料，有助于获得监管部门对于新药临床试验（investigational new drug, IND）的批准。例如：2022年5月，CDE基于一项IIT的研究结果，批准伊基仑赛注射液用于治疗视神经脊髓炎谱系疾病的临床试验申请^[7]^。

IIT可以作为IST的补充，为新药的IND与NDA提供支持性证据，利用IIT研究上市后药物拓展新的适应证还可能降低新药研发的费用^[8]^，有助于控制药费、降低疾病负担。因此，应当提倡开展高质量IIT助力新药研发。

## 3 存在的问题及挑战

### 3.1 研究数量多、规模小、质量参差不齐

国内外的IIT普遍存在研究数量多、但规模小、质量参差不齐，难以获得高水平研究成果的情况^[8-11]^。2021年发表在《英国医学杂志》的一项研究指出，2008年-2019年，中国大陆机构发表了2, 000多项他汀类药物治疗冠心病的IIT研究。这些研究只是简单重复了药物已知的疗效，没有得出任何新的有价值的研究结果^[12]^。国内一些针对IIT研究质量的调查也发现IIT研究缺少成熟的研究团队和充足的经费支持，在研究方案设计、知情同意书签署、研究过程中方案依从等方面均与IST有明显差距。特别是，由于缺乏系统的监查、稽查与核查，研究数据的可靠性、完整性存在一定的不确定性，研究结果递交监管部门时会面临质疑，甚至导致研究失败。

### 3.2 研究者的方案设计能力和研究管理能力不足

部分IIT研究设计存在科学性问题甚至存在明显的伦理挑战，研究结果难以用于支持NDA，主要原因是研究者的方案设计能力不足。大多数IST研究目的明确，申办者企业有成熟的研究设计团队，且方案经过了CDE的审核，保证了研究设计的科学性。IIT的研究者为了解决临床中发现的问题而设计研究，但由于临床问题凝练不足、文献检索不充分、既往研究数据收集不完整，导致研究目的不明确或立项缺少充分论证和依据。部分IIT的研究者缺乏临床研究的基本素养，对于方案设计中的重要科学性要素缺乏把控能力，导致研究的对照设置、受试者人群选择、研究药物的剂量、疗程、联合治疗方案的确定、主要疗效指标、样本量、统计学方法等关键设计缺乏科学性和严谨性。例如，肿瘤IIT研究中大量的单臂、联合设计可能损害患者获益并具有额外不可控风险。

IIT的研究者还存在研究管理能力不足的问题。主要研究者在日常繁忙的临床工作之余常常无暇顾及临床研究，其主要研究程序的执行者和数据的收集者往往研究经验匮乏，合规意识不强，导致研究数据收集在及时性、准确性、完整性等方面存在缺陷。IIT研究中研究者风险意识薄弱，也没有完整有效的风险控制体系，个别研究者对于受试者的保护关注不足，在研究过程中，对于不良事件的处理、报告不及时，退出终止研究的标准掌握不严格，应当承担的受试者补偿不及时支付等情况也时有发生。

### 3.3 医疗机构对于IIT的立项管理标准与IST不同

制药企业发起的临床试验，经药监部门批准后方可实施，研究过程及研究结果也受到严格的监管。而研究者发起的临床研究，卫生行政主管部门尚未建立一整套行之有效的监管策略，落实到医疗机构内对于IIT的监管往往与IST标准不同。

目前医疗机构对于IIT的管理大致分为两种模式：IIT与IST管理标准相同或管理标准不同。IIT与IST同质化管理可以保证对IIT的管理水平，但由于管理资源有限或者增加临床研究数量等需要，目前采用这种管理模式的医疗机构不多，IIT与IST管理标准不同的情况更为常见。IIT与IST由不同部门管理时往往IIT的管理更松，即便IIT与IST由同一部门管理，也有不少是资源向IST倾斜，而对IIT的重视程度不高，仅仅依靠主要研究者负责制，对于IIT的立项及研究质量、数据安全、风险控制等缺少实质性管理，立项管理和学术审查形同虚设，有“失监管”甚至“故意放水”的现象。更有甚者开展一些没有明确的研究目的、不以解决临床问题为导向，而是制药企业出于占领市场的需要，打着IIT的幌子进行市场营销的项目^[13]^。

### 3.4 伦理审查能力有待提高

根据目前的法规要求，开展IIT的医疗机构必须设置伦理审查委员会，伦理委员会的规范性建设虽然有进步，但IIT的伦理审查能力不足仍然是目前比较突出的问题。IST伦理审查规范较为完善、审查严格、文件完整。即便如此，国产抗肿瘤药物信迪利单抗在美国FDA申报上市被拒绝的原因体现出了我国临床研究的伦理审查仍需进一步加强。与IST相比，IIT伦理审查工作尚处于起步阶段，调查显示IIT的伦理审查较为放松、更容易被批准^[14]^。部分伦理委员会在初始审查中不能识别研究设计中挑战伦理原则的问题，例如：对照组的选择疗效已明显劣于患者可接受的可获及治疗、研究过程中对于不再获益的受试者没有设置合理的退出机制，研究的样本量或统计学方法不合理而影响疗效和安全性评估的可靠性等。而与初始审查相比，IIT的伦理跟踪审查则更不规范甚至缺失^[15]^。IIT研究的不良事件处理、受试者补偿等往往未被伦理委员会严格监管。由于存在潜在的伦理风险，利用IIT研究结果支持新药研发上市也将面临一定的挑战。

### 3.5 经费不足

在我国IIT经费来源大致有三类：政府设立的纵向课题；企业或基金会赞助；研究者自筹。我国医学研究资助体系中，大多数针对基础研究，对IIT的资助非常有限，而临床研究的预算又往往较高。二者之间严重的不平衡整体呈现出IIT经费不足的现象。由于研究经费的不足，在研究设计上不得不做出一些妥协，例如减少例数、删减必要的检查内容或降低检查的频率、使用替代终点以缩短研究时间等。设计上的改变甚至将会影响研究结果的质量。经费不足还会导致实施过程中没有足够的团队保证数据质量，由于缺少交通补偿可能导致受试者脱落，没有必要的补偿机制或赔偿内容不明确甚至带来医患纠纷等^[16]^。

## 4 对策及建议

### 4.1 国家层面加强对IIT的监管

尽管卫健委颁布的《医疗卫生机构开展研究者发起的临床研究管理办法（试行）》已经开始在北京、上海、广东、海南四个省市进行试点，规范开展IIT的相关制度、政策特别是管理细则仍不明确，国家层面的监督管理还亟待加强。应当尽快通过试点总结出一套可普遍推广的IIT管理要求，并且借鉴药监局临床试验数据核查的经验，开展IIT的质量监督检查，提升医疗机构及研究者对IIT的重视程度。对于没有管理能力的医疗卫生机构和研究者，可以限制其开展用于支持新药研发的IIT。

### 4.2 医疗机构加强对于IIT的管理

IIT由于干预措施、经费来源、研究者能力等不同导致项目的风险存在差异，管理难度也随之加大。医疗机构应当成立专门的管理部门，制定相应的管理制度规范，对研究者发起的临床研究实施分类、严格、规范的管理。

支持新药研发的IIT应按照IST要求进行相应的立项前的学术审查。学术委员会的审查范围和审查能力应当与开展的研究相匹配。除了本机构内的学术委员会，可以邀请机构外的同行评议，还可以在立项前与CDE进行沟通交流，重点评估开展研究的前期基础是否充分，研究设计是否合理等。

支持新药研发的IIT必须严格遵守药物临床试验质量管理规范(good clinical practice, GCP），医疗机构应当使用与IST相同的质量管理体系，必要时可以请独立的第三方稽查协助提升研究质量。质控重点是可能严重影响研究结果的关键环节与关键数据，包括研究中纳入不符合入排标准的受试者、合并使用禁用药物、关键访视或重要观察指标的缺失等。也要关注可能降低数据完整性和准确性的反复大量出现小的方案违背，例如检查漏做、受试者漏服药物、访视超窗等。研究者既往研究经验少、病例登记和随访的支持系统不完备、入组病人数量非常多或非常少的项目，属于研究质量风险高的项目，还要予以特别关注。

### 4.3 提升伦理审查能力

IIT的伦理审查应当实现与IST的同质化管理，有条件的医疗机构建议由同一个伦理委员会开展对IST和IIT的审查。项目比较多或确有必要分别由不同的伦理委员会审查时，应当采用统一的管理体系，开展相同的培训，并尽可能在不同伦理委员会中有一定的委员重叠，以保证所有伦理委员会的管理水平、审查能力和审查标准一致。

用于支持新药研发的IIT，初始审查也应当就研究的科学社会价值、临床前研究和已完成的临床研究的基础、受试者人群的选择、研究方案设计、研究的风险获益以及受试者保护等因素进行充分审查。还必须根据风险程度落实研究过程中的跟踪审查，特别对方案、知情同意书的修正、研究过程中的严重不良事件、方案偏离/违背等进行严格审查，以确保受试者的权益和研究结果的可靠，足以支持未来的注册申请。

研究相关费用的支付及研究相关损害的诊疗和补偿是受试者权益中的重要部分。IIT经费来源不同，存在经费支持不足的风险，伦理委员会应切实在审查中履行受试者保护的职责。初始审查对于明显经费不足以支持开展的研究，不予批准。跟踪审查时，重点关注对于研究过程中发生的严重不良事件的处理及应当支付受试者的补偿的落实情况。

### 4.4 提升研究者的能力

首先需要提升研究者的责任意识，研究者是临床研究的核心，特别是在用于支持新药研发的IIT中，研究者除了应当承担研究的实施、受试者的管理、研究数据的收集和结果的整理等责任，还需要承担申办者的部分甚至全部职责，包括设计研究方案、监管研究质量、报告不良反应等。

应当定期组织研究者培训，培训内容应当涵盖研究方向梳理和科学问题凝练的能力、研究方案设计的能力、基本的流行病和统计学方法、临床研究实施的法规和伦理学要求、相应品种注册临床试验的技术指导原则等。培训应当结合实际需要、要有针对性，还要反复多次开展，培训后应当以多种形式检查培训效果。

研究型医院可以考虑搭建IIT的支撑平台，提升对研究者的支持能力。配备研究型病房、中心药房、相应的信息化系统等，并由专职的研究支持人员为研究者提供研究资料检索、方案设计、统计分析计划、数据档案管理、受试者管理等服务。必要时充分和制药企业、监管机构以及其他医疗机构建立多方联系，共同协作管理研究风险。

### 4.5 保障研究经费充足

IIT用于支持新药研发，当研究者的研究方向与企业的研发策略不相吻合，不能从制药企业得到全额的经费支持时，则需要更多源的经费保障。由于历史、制度、文化等原因，我国来自慈善基金、信托基金和个人捐款用于临床研究的还非常少，应当在监管政策、舆论导向等方面进一步发力，引导学术组织、社会团体、基金会和个人等社会资金投向临床研究。广泛接受各种基金的资助，既可以有效缓解政府投入的不足，也可以规避由于过渡依赖企业资助，导致研究立题、过程实施、结果发布等环节受企业意愿的影响产生的偏倚。无论经费来源于哪一方，都需要严谨的合同条款约定各方的责任和权利，也需要严格遵守利益冲突回避制度，从而保证经费的使用合法、合规。

综上所述，IIT对于支持新药研发有重要价值，但IIT数量多、项目复杂、管理难度大。需要监管部门、申办方、研究机构、伦理委员会和研究者共同提高对于IIT用于支持新药研发作用的认识，深入了解研究机构、研究项目、研究者的特点及管理复杂性，有针对性地采取解决措施，积极促进高质量IIT的开展，以期更多高水平的研究成果用于解决未被满足的临床需求，推动新药研发和上市。
